# Conservative Management of a Distal Humerus Spiral Fracture Sustained During Arm Wrestling: A Case Report and Literature Review

**DOI:** 10.7759/cureus.42466

**Published:** 2023-07-26

**Authors:** Basil Jalamneh, Hamza Salim, Leen Sabbooba, Ameed Taher, Nadeem Khayyat, Mohammad M Jaber, Mazen Abdalla

**Affiliations:** 1 Faculty of Medicine and Health Sciences, An-Najah National University, Nablus, PSE; 2 General Practice, Palestinian Ministry of Health, Ramallah, PSE; 3 Radiology, Jenin Government Hospital, Palestinian Ministry of Health, Jenin, PSE; 4 Orthopaedics and Traumatology, Najah National University Hospital, Nablus, PSE

**Keywords:** cast stabilization, external reduction, biomechanical, conservative management, spiral fracture, humerus fracture, arm wrestling

## Abstract

Arm wrestling is a popular recreational activity that involves intense and repetitive muscular contractions of the upper extremity. Arm wrestling can result in various musculoskeletal injuries, including bone fractures. Humeral shaft fractures, particularly spiral fractures, are the most common fractures resulting from arm wrestling. Here, we present a case of a 25-year-old male patient who sustained a distal humerus spiral fracture during an arm-wrestling competition. The patient was managed conservatively with external reduction, cast stabilization, and physiotherapy with good outcomes. Despite initial angulation and rotation, the fracture healed well, and the patient regained full function of his arm within eight weeks. This case highlights the biomechanical aspects and risk factors of this type of fracture during arm wrestling and the value of protective measures such as proper technique, training, and protective equipment. It also highlights the potential for conservative management in such cases.

## Introduction

Arm wrestling, a widely practiced sport among professionals as well as amateurs, is generally considered safe when executed correctly. However, it poses potential risks, mostly among amateurs with flawed techniques [1].

While fractures of the distal humerus are not frequently associated with arm wrestling [2], they do occur, with distal humerus spiral fractures being the most prevalent type [3].

Arm wrestling can also result in various musculoskeletal injuries, including other bone fractures, dislocations, ligament injuries, and muscle strains. In addition to humeral fractures, unusual fracture patterns, such as solitary radial shaft fractures, scapular neck fractures, radial neck fractures, and extra-articular olecranon fractures, have also been reported in arm wrestlers, particularly in males. Soft tissue injuries, such as ligamentous and tendinous injuries throughout the upper limb, have also been documented [4].

In their review, Moloney et al. reviewed 158 cases of arm-wrestling injuries reported in the literature [4]. Humeral shaft fractures constitute 68% of these cases (n=108 cases). Kiyohisa Ogawa et al. reviewed 153 cases of humeral fractures sustained during arm wrestling [5]. All fractures were spiral, and 90% of the fractures were located at the distal part of the junction of the middle and distal part of the humerus. There is an obvious gender bias toward males for this type of injury [4-5]. An amateur male in his twenties is the typical presentation. Kiyohisa Ogawa et al. reported the patients' mean age as 26.1 ± 9.0 years (range, 10-63 years) [5].

There are no data that show the superiority of any treatment modality over the other; half of the cases opted for conservative management [4]. Besides, a favorable clinical outcome of conservative management of humeral fractures is seen despite the mechanism of the fracture [2].

Here, we present a case of a distal humerus spiral fracture in a 25-year-old non-athletic man that was sustained during an arm-wrestling activity. It was managed conservatively with good outcomes.

## Case presentation

**Figure 1:**
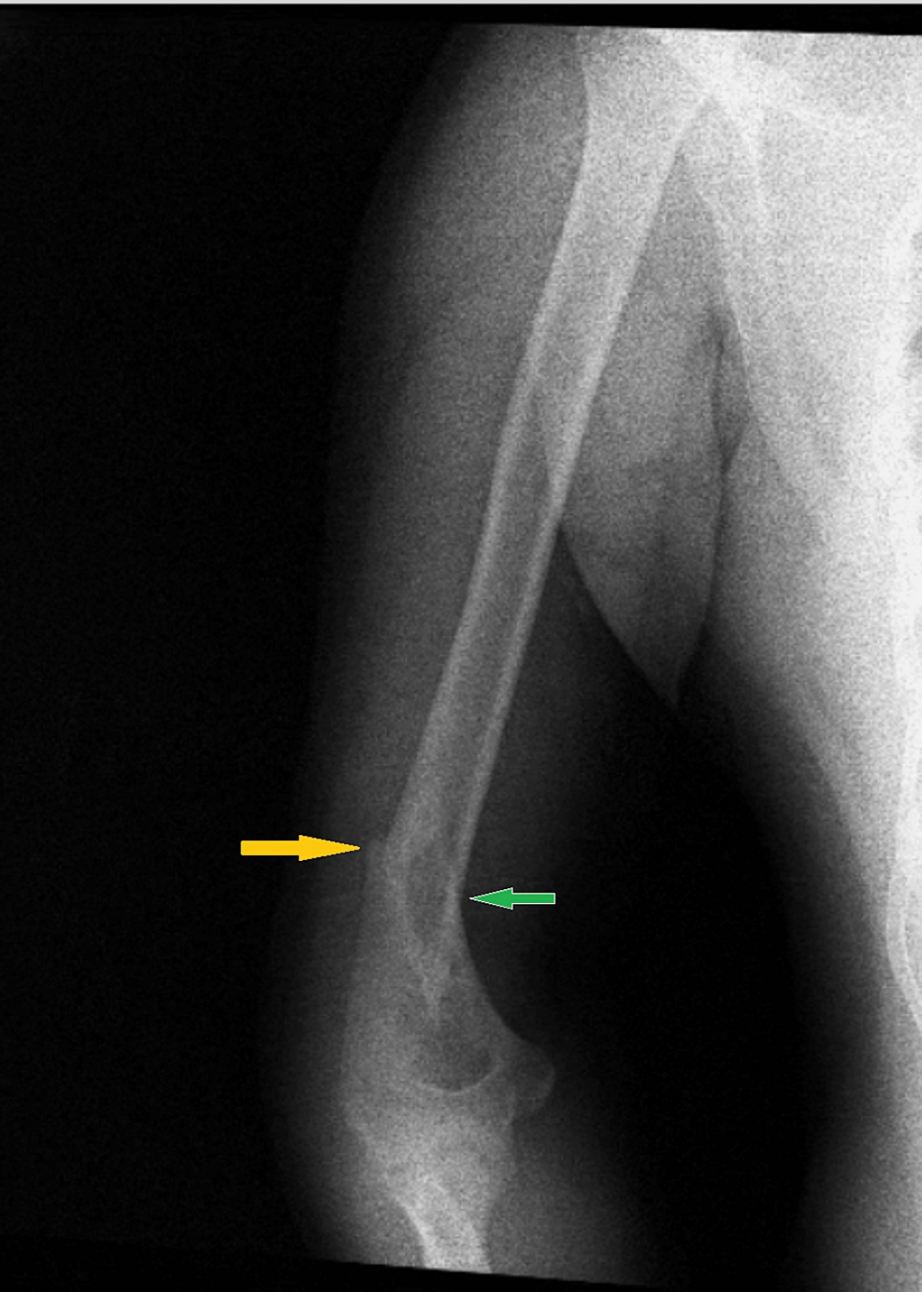
An anterioposterior radiograph view of the humerus showing the displaced distal humerus spiral fracture (yellow arrow) Rotation and angulation (green arrow) displacement is evident in the image shown.

**Figure 2:**
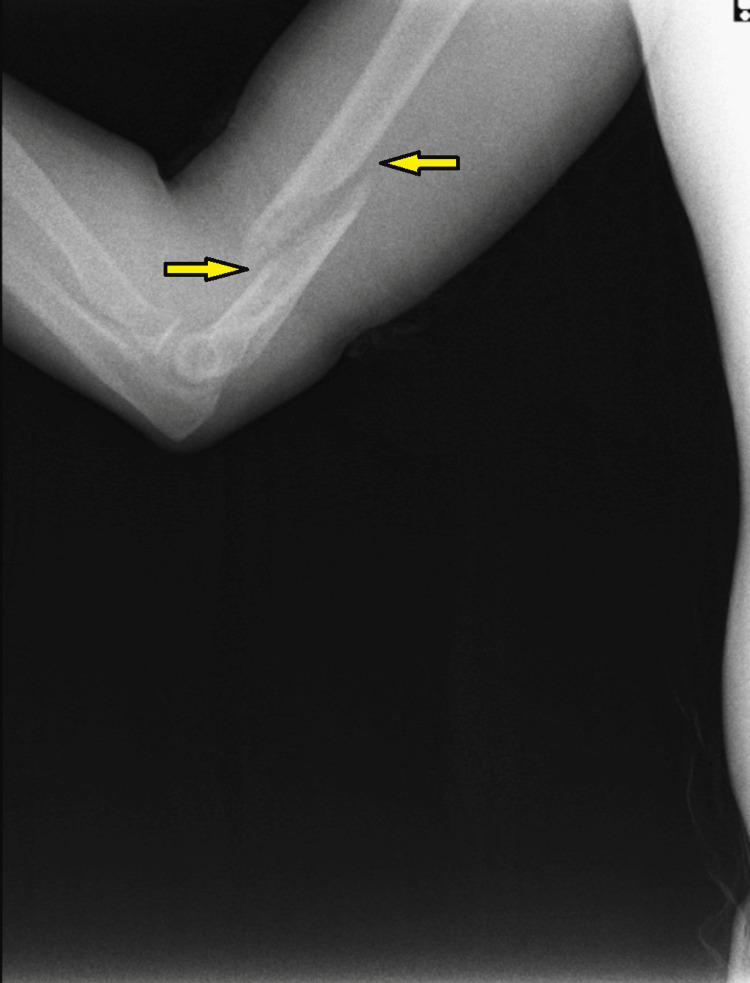
Lateral view radiograph of the humerus showing the displaced distal humerus spiral fracture (yellow arrows) Rotational displacement is evident in this image.

A 25-year-old male patient presented to the emergency department due to an injured right arm during an arm-wrestling activity. The patient was an amateur and right-handed, and his opponent was a young adult male of an equal build and power. The patient reported that he had felt his arm snipping out of its place with a loud popping sound and that his wrestling hand was no longer connected to his arm. This occurred after a prolonged wrestling bout and during the winning (offensive) phase when he applied maximum force with his wrist flexed. He reported no pre-match warm-up. Initially, there was no pain, as he kept his elbow on the table. The pain then increased gradually in the elbow region and was exacerbated by any slight movement of the elbow, he rated the pain to be 7 out of 10. The pain was associated with nausea. He had no history of bone disorders or bone fractures and used intranasal steroids occasionally for allergic rhinitis management. He did not take any other medications or supplements. He was an E-cigarette smoker, did not drink alcohol, and his BMI was 29. There is no family history of bone disorders. On physical examination, the patient was holding his arm in a flexed, adducted, and internally rotated position, a mild elbow swelling without obvious hematoma was noted and there was tenderness on the lateral side of the humerus a few centimeters above his elbow joint. He had an intact neurovascular examination with stable vital signs. Initial X-rays showed a spiral fracture in the humeral shaft distally, as shown in Figures 1, 2.

The patient underwent external reduction once again, which resulted in an acceptable angle and rotation, and his fracture was stabilized with a cast in a U-shaped hanging position (elbow at 90 degrees).

**Figure 3:**
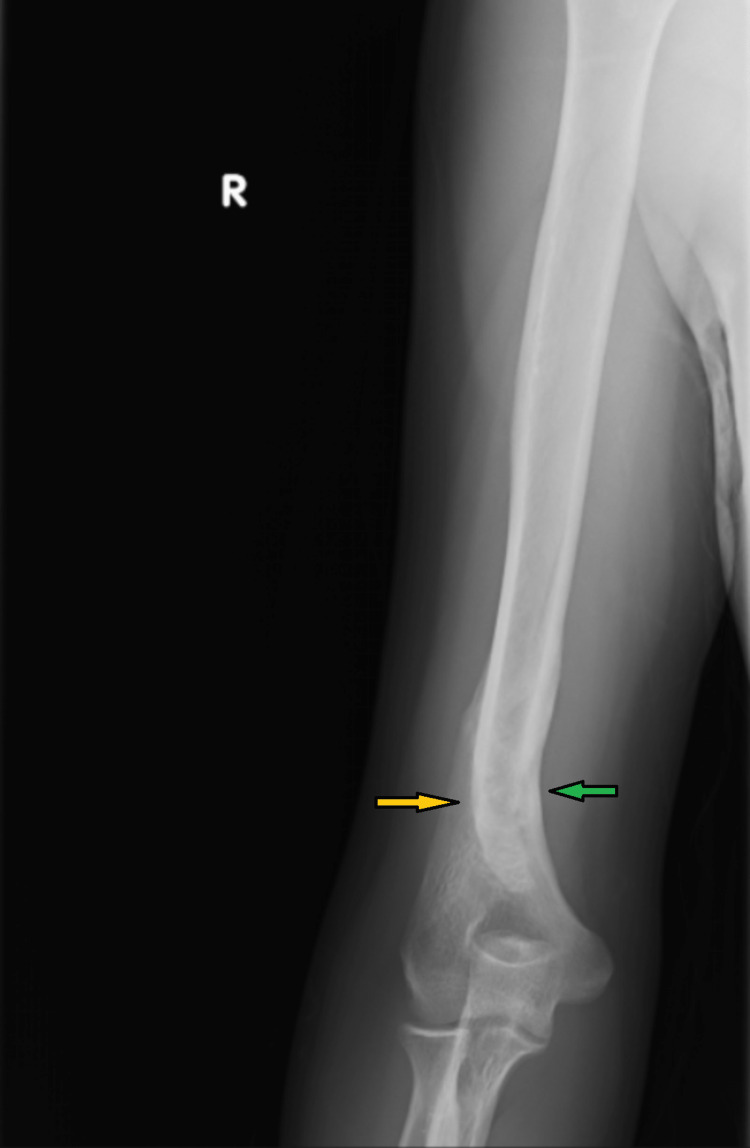
An anterioposterior view humerus radiograph showing a well-healed ossified distal humerus fracture (yellow arrow) An acceptable angulation deformity is seen (17 degrees) (green arrow).

**Figure 4:**
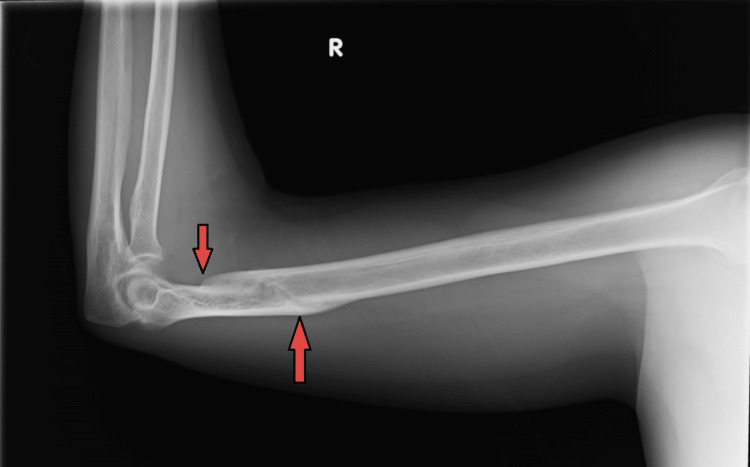
Lateral view humerus X-ray showing a well-healed, ossified distal humerus fracture

Six weeks later, the plaster was replaced with a full fiber cast that only covered the arm while freeing the elbow. His arm remained hanging. By that time, he began to move his elbow with simple active motion, and he was able to perform some usual daily activities in addition to returning to his job as a teaching assistant. Ten weeks after the initial accident, we removed the cast, and he started moving his elbow more freely. The patient had some stiffness in the elbow as well as an incomplete extension. However, pronation and supination, as well as neurologic examination, were all normal. In the eighth week, the patient regained all functions of his arm and started home physiotherapy that focused mainly on increasing the extension range of the elbow and strengthening the biceps muscle. Physiotherapy was guided by the specialty advice and progression was monitored by the orthopedic surgeon during follow-up visits. He started elbow training with flexion and extension using a light weight of 1 kilogram increasing the weight gradually. He also trained his shoulder with a wide range of shoulder exercises such as flexion, extension, abduction, rotation, press, and isometric exercises. In the end, he was able to lift heavy weights of 15-20 kilos for biceps, triceps, and deltoid muscle strengthening. Now after seven months of follow-up, he has a complete extension of the elbow, and he regained the full power of the arm and the full range of movements with a slight varus deformity. Aside from a minor cosmetic issue, the deformity poses no limitation to the biomechanical functions of his arm. He continued weightlifting exercises. X-rays showed a well-healed fracture with acceptable displacement (Figures 3, 4). 

## Discussion

Humeral shaft fractures, particularly spiral fractures, are the most common fractures resulting from arm wrestling. It is believed that torsional forces generated by the subscapularis, pectoralis major, and latissimus dorsi in the shoulder (internal rotator shoulder muscles), which are resisted by external rotation or another counterforce from the opponent, eventually lead to torsional strain, bending force, and axial compression on the humerus, resulting in a spiral fracture [4-6].

While humeral shaft fractures from arm wrestling are rare, several risk factors might increase the possibility of such an injury. They include biomechanical aspects, such as the posture of the torso and arms during arm wrestling, and the anatomical qualities of the bone such as bone density (e.g., osteoporosis). Humeral fractures in arm wrestling may also be caused by improper technique, insufficient training, muscular hypertrophy, and lack of effective motor control. Amateurs are more prone to this injury, as they tend to stabilize the arm in the shoulder (glenohumeral joint) [1]. Arm wrestling with the usage of anabolic steroids has been linked to humeral shaft fractures. The utilization of anabolic steroids can lead to unfavorable changes in the cortical thickness of bones and the power of muscles, potentially serving as a predisposing element for arm-wrestling fractures [3]. Prior research has indicated that alcohol intake and the absence of warm-up routines can also be considered potential risk factors [7]. Guidance for arm-wrestling matches has been recommended to limit the incidence of these fractures. These rules include choosing participants of similar height and weight, avoiding abrupt ends, and using proper techniques. Moreover, the use of protective equipment, such as braces or elbow pads, can minimize the risk of fractures and dislocations [3,8-10].

**Figure 5:**
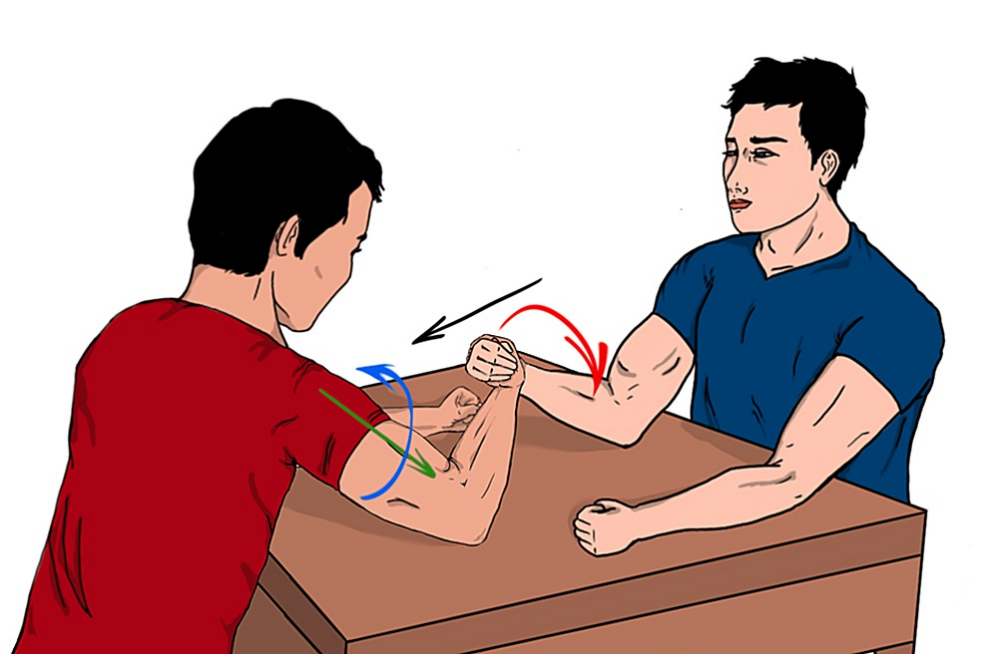
An illustration of the arm-wrestling bout The contestant with a red shirt applied the maximum internal rotation force with his elbow flexed in addition to a flexion force toward the chest that eventually lead to the fracture. Red arrow: external force (external rotation), blue arrow: internal rotation force (pectoralis major and subscapularis forces), green arrow: axial load resulted from pulling toward his chest (biceps and brachialis forces) Image: authors' own creation

**Figure 6:**
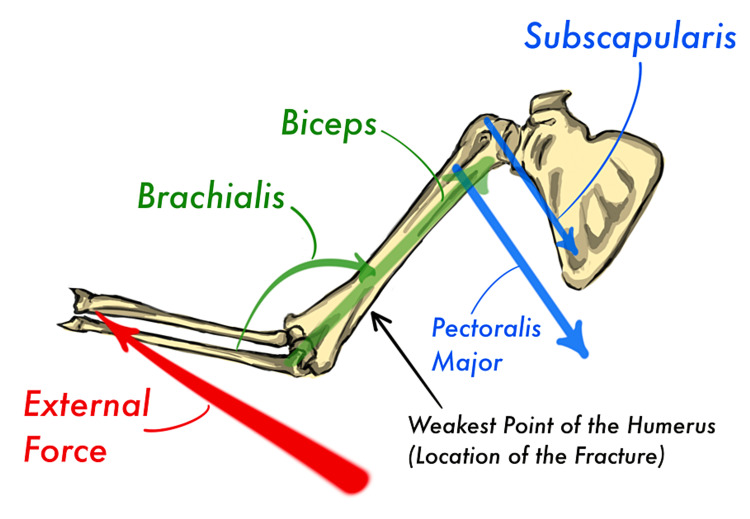
An illustration of counter-forces in the fractured arm Red arrow: external rotation force, blue arrows: internal rotation force, green arrows: axial load Image: authors' own creation

The incident described was witnessed and analyzed by two medical doctors who are co-authors of this case. Our objective is to formulate a theoretical basis for the fracture trigger. The arm-wrestling bout took place on a rough wooden table and both competitors were seated, leading to a slightly wider elbow angle than usual. The match went on for approximately three minutes until both individuals were exhausted. Nevertheless, they persisted in applying equal force with their arms positioned in the middle. Subsequently, the participant exerted maximum force while twisting his wrist and pulling the other's arm toward his chest, causing elbow flexion that ended in his fracture. During this maneuver, their torso, shoulder, and elbow remained immobile. A biomechanical assessment of the event revealed excessive internal rotation force and forceful elbow flexion. The technique performed, and the resulting forces are illustrated in Figures 5, 6.

BY Low et al. reported favorable outcomes in arm-wrestling competitions held in Singapore when certain protective measures were implemented such as restricting the match duration to three minutes and maintaining proper posture during the contest [11]. However, they also documented five cases of humerus fractures related to arm wrestling when participants ignored these measures. We contend that the patient's injury was due to a combination of these factors, along with their flawed technique.

Presently, there are no universal time limits for arm wrestling matches in professional games. We advocate for further research examining the correlation between match duration and injury incidence. Furthermore, we recommend that arm wrestling tables be designed with appropriate dimensions to accommodate both seated and standing positions.

Surgery and conservative treatment options are available for humeral shaft fractures caused by arm wrestling. Three of six patients in a study of arm-wrestling-related humeral shaft fractures opted for open reduction and internal fixation while the other three received non-operative care. Mobilization for patients undergoing internal fixation could begin as early as one month after surgery, compared to more than two months for those undergoing conservative treatment. All patients treated conservatively had residual varus deformity at the fracture site, whereas all patients treated with internal fixation had no residual deformity and regained full range of motion [3].

The treatment strategy for humeral shaft fractures should consider the patient's age, general health, and level of activity, as well as the location and severity of the fracture. More severe fractures, those with significant displacement, or those with evidence of vascular or nerve damage may need surgical intervention. Patients who are younger and more physically active and who are eager to resume normal activities may also benefit from this approach. However, surgical treatment carries risks, including the possibility of infection, complications from anesthesia, and failed implants. On the other hand, patients with less severe fractures or who are not good surgical candidates may benefit from conservative treatment. The risks of surgery can be avoided, making this a viable alternative for patients who would rather avoid or postpone the invasive procedure. The choice between surgical and nonsurgical treatment should be made in consultation with an experienced orthopedic surgeon and after carefully considering the patient's unique needs and goals [3,6,12,13]. Moreover, there is either a lack of evidence or inadequate evidence from randomized or quasi-randomized controlled trials to ascertain the effectiveness of surgery and which specific surgical interventions are suitable for treating various types of distal humerus fractures [14]. In this case, the uncomplicated presentation of the patient's fracture with no neurovascular injury and the absence of a wedge fragment as well as the patient's enthusiasm to adhere to programmed physiotherapy favored the conservative management approach.

## Conclusions

Arm wrestling has the potential to cause a variety of musculoskeletal injuries, including bone fractures, dislocations, ligament tears, and muscle strains. Arm wrestling is an uncommon cause of humeral fracture. However, distal humerus spiral fractures are the most prevalent type of fracture in this sport. These injuries can occur due to non-adherence to protective measures, poor technique, insufficient training, muscle hypertrophy, and ineffective motor control. The biomechanical causes and some risk factors in this patient's fracture match the ones reported in the literature. There are both surgical and conservative treatment options. Less severe fractures may be treated with plaster or splint casts, whereas more serious fractures need open reduction, internal fixation, and immobilization. The treatment plan should consider the patient's age, general health, degree of activity, fracture site, and severity. With adequate treatment, patients may have favorable results and restore full arm function. Participants in arm wrestling should adhere to prescribed rules and guidelines to reduce the frequency of fractures and injuries. Our case is an example of the optimum outcomes that resulted from a conservative management approach due to adherence to the physiotherapy program and its proper execution as well as close follow-up. The patient has regained full function and range of motion of the arm with no significant residual deformity.

## References

[1] Kruczyński J, Jaszczur Nowicki J, Topoliński T (2012). Radiological and biomechanical analysis of humeral fractures occurring during arm wrestling. Med Sci Monit.

[2] Mayfield CK, Egol KA (2018). Humeral fractures sustained during arm wrestling: a retrospective cohort analysis and review of the literature. Orthopedics.

[3] Pande KC, Nishat N, Afzal S, Ishak L (2021). Humeral shaft fracture sustained during arm wrestling with review of factors contributing to its causation. Malays Orthop J.

[4] Moloney DP, Feeley I, Hughes AJ, Merghani K, Sheehan E, Kennedy M (2021). Injuries associated with arm wrestling: a narrative review. J Clin Orthop Trauma.

[5] Ogawa K, Yoshida A, Matsumura N, Inokuchi W (2022). Fractures of the humeral shaft caused by arm wrestling: a systematic review. JSES Rev Rep Tech.

[6] Şahin T (2020). Arm wrestling related injuries: a literature review. Int Arch Orthop Surg.

[7] Shen J, Yu P, Yang R (2023). Clinical characteristics, mechanism, and outcome of humeral shaft fractures sustained during arm wrestling in young men: a retrospective study. Orthop Surg.

[8] Cordey J, Grütter R, Johner R (2000). The mechanical strength of bones in torsion application to human tibiae. Injury.

[9] Nordin M, Frankel VH (2012). Basic Biomechanics of the Musculoskeletal System. https://shop.lww.com/Basic-Biomechanics-of-the-Musculoskeletal-System/p/9781975141981.

[10] Ogawa K, Ui M (1997). Humeral shaft fracture sustained during arm wrestling: report on 30 cases and review of the literature. J Trauma.

[11] Low BY, Lim J (1991). Fracture of humerus during armwrestling: report of 5 cases. Singapore Med J.

[12] Schwab TR, Stillhard PF, Schibli S, Furrer M, Sommer C (2018). Radial nerve palsy in humeral shaft fractures with internal fixation: analysis of management and outcome. Eur J Trauma Emerg Surg.

[13] Shao YC, Harwood P, Grotz MR, Limb D, Giannoudis PV (2005). Radial nerve palsy associated with fractures of the shaft of the humerus: a systematic review. J Bone Joint Surg Br.

[14] Wang Y, Zhuo Q, Tang P, Yang W (2013). Surgical interventions for treating distal humeral fractures in adults. Cochrane Database Syst Rev.

